# Macromolecular and Solution Properties of the Recombinant Fusion Protein HUG

**DOI:** 10.1021/acs.biomac.2c00447

**Published:** 2022-07-25

**Authors:** Paola Sist, Antonella Bandiera, Ranieri Urbani, Sabina Passamonti

**Affiliations:** † Department of Life Sciences, University of Trieste, Via Giorgieri 1, Trieste I-34127, Italy; ‡ Department of Chemical and Pharmaceutical Sciences, University of Trieste, Via Giorgieri 1, Trieste I-34127, Italy

## Abstract

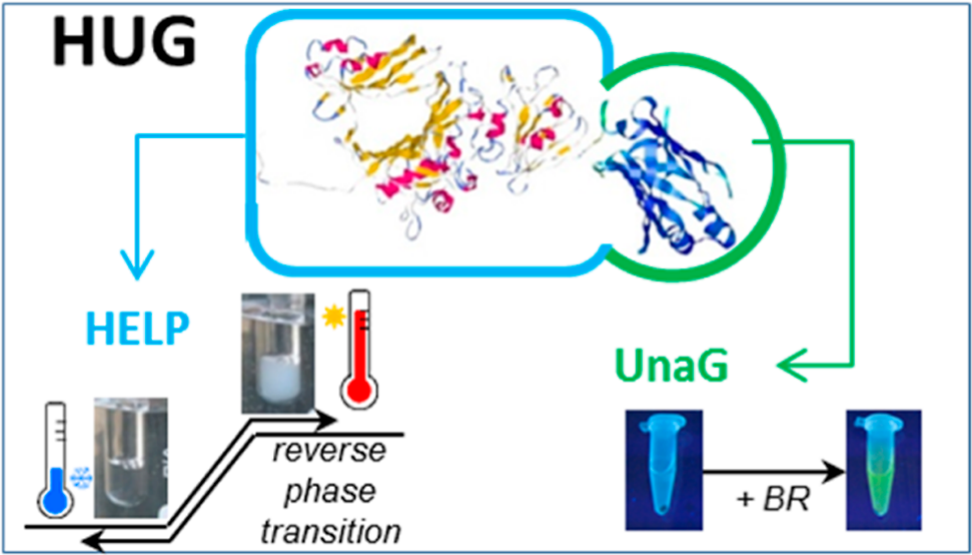

The recombinant fusion protein HELP-UnaG (HUG) is a bifunctional product that exhibits human elastin-like polypeptide (HELP)-specific thermal behavior, defined as a reverse phase transition, and UnaG-specific bilirubin-dependent fluorescence emission. HUG provides an interesting model to understand how its two domains influence each other’s properties. Turbidimetric, calorimetric, and light scattering measurements were used to determine different parameters for the reverse temperature transition and coacervation behavior. This shows that the UnaG domain has a measurable but limited effect on the thermal properties of HELP. Although the HELP domain decreased the affinity of UnaG for bilirubin, HUG retained the property of displacing bilirubin from bovine serum albumin and thus remains one of the strongest bilirubin-binding proteins known to date. These data demonstrate that HELP can be used to create new bifunctional fusion products that pave the way for expanded technological applications.

## 1 Introduction

The development of biopolymers based on natural protein structures holds great potential in the field of biotechnological and biomedical applications. Using recombinant DNA technology, a subclass of elastin-like polypeptides has been developed and studied in our laboratory, the human elastin-like polypeptides (HELPs), whose structure is based on a repeating hexapeptide motif (Val-Ala-Pro-Gly-Val-Gly) found in human elastin.^1^ HELP (*M*_w_ = 45 kDa) is encoded by a synthetic gene consisting of eight elastin-like blocks and a unique restriction site that allows in-frame cloning of any protein sequence of interest.^2^ HELP is subject to a reversible phenomenon called inverse transition temperature (ITT). At temperatures below the ITT, the biopolymer is soluble in aqueous solutions because the HELP monomers undergo favorable interactions with the solvent and are mainly in a disordered and fully hydrated state. In contrast, at temperatures above the transition temperature, these chains exhibit a decreased solvent-accessible surface area and an increase in interchain contacts stabilized by hydrophobic interactions, favoring their association and the formation of an amorphous solid phase.^3^

Several HELP C-terminal fusion proteins have been described that have been shown to retain the biological activity of the functional domain.^4^ Among them, the fusion with the UnaG protein was particularly interesting since despite the size of this domain (139 aminoacids) with respect to the HELP moiety (∼500 aminoacids), the final construct retained the HELP phase-transition properties as well as bilirubin (BR) binding and subsequent fluorescence capacity.^5^ Moreover, this capacity was retained after enzymatic crosslinking, resulting in a functional matrix.^6^ This prompted us to further investigate the macromolecular and solution properties of this recombinant fusion protein. Quantitative determination of the macromolecular and physicochemical properties of HUG in solution provides the basis for optimizing the purification of the HUG polymer from bacterial expression systems. Understanding the behavior of HUG in dilute solutions and aggregation phenomena as a function of temperature are important insights for optimizing crosslinking processes. Thermodynamic and spectroscopic techniques can be used to elucidate the reverse-transition property of HUG.^6^ In addition, we can use fluorometric techniques to characterize the binding capacity of the HUG biopolymer, a property that could be affected by the HELP primary structure added to the UnaG domain. The presence of the HELP domain could alter the environment of UnaG, leading to a change in the structure of UnaG and a change in its functionality.

The possibility of using the UnaG domain bound to HELP for the determination of BR in biological fluids (*e.g.*, animal blood) requires an evaluation of the affinity constant in the presence of albumin. In human blood plasma, BR is present in the range of 3–15 μM.^7^ BR is reversibly bound to albumin, whose average concentration is about 600 μM, which prevents it from passing the intact blood–brain barrier.^7,8^ Many authors reported that the binding of BR to albumin occurs at only a few binding sites.^8,9^ Remarkably, there are at least three types of binding sites for bilirubin to albumin. The strongest and most important binding site for BR (subdomain IIA, amino acid positions about 190–300) has a very high binding constant, *K*_a_ ∼ 10^7^ L·mol^–1^, and is considered the specific site.^10^ In addition, spectroscopic studies and measurements of peroxidase oxidation rates have shown that other secondary sites (IB and IIIA) have about a 10-fold lower binding affinity, *K*_a_ ∼ 10^3^–10^6^ L·mol^–1^.^10,11^ Many compounds and drugs (predominantly anionic molecules and aromatic structures that are poorly water-soluble) have been screened for BR displacing effects on the BR-albumin complex and have shown competitive binding to the high-affinity bilirubin site of albumin.^12−14^ However, in our work, we aim to evaluate the ability of the UnaG probe inserted into HUG to subtract BR from albumin by forming a stable bond with the pigment.

The main objective of this study was to investigate the macromolecular and solution-specific properties of HUG to characterize relevant differences with respect to HELP and UnaG. This study investigated the extent to which a protein domain fused to the C-terminus of HELP affects its physicochemical properties. Finally, a detailed BR-binding process study was performed to verify that HUG retains the binding properties of UnaG in the presence of albumin.

## 2 Materials and Methods

### 2.1 Reagents

The BR used was purchased from Sigma-Aldrich (Lot. 031M1429V #B4126; Sigma-Aldrich), and it has a major proportion of IXα-isomer (91.49%) with trace amounts of IIIα (4.02%) and XIIIa (3.33%). Dimethyl sulfoxide (DMSO), sodium phosphate dibasic (Na_2_HPO_4_), sodium phosphate monobasic (NaH_2_PO_4_·H_2_O), sodium chloride (NaCl), and hydrochloric acid (HCl) were all of analytical grade and obtained from Sigma-Aldrich.

### 2.2 HUG Biosynthesis and Purification

The synthesis and purification of HUG (HELP-UnaG) have been described in detail previously.^6^ Briefly, the HUG biopolymer was obtained using the synthetic gene of the HELP polypeptide fused to the 139 amino acid coding sequence of the bilirubin-binding protein UnaG. The fusion product was also expressed in *E. coli* and purified using the HUG inverse phase-transition properties. The recombinant products (approximately 2 g L^–1^ protein) were analyzed by both sodium dodecyl sulfate polyacrylamide gel electrophoresis (SDS-PAGE) analysis and ultraviolet spectroscopy at λ = 250–350 nm. The purified product was lyophilized for long-term storage and checked for purity by SDS-PAGE and UV–vis spectroscopy (Figure S1 in the Supporting Information).

### 2.3 Secondary Structure Prediction

The average hydropathy value (GRAVY) for a protein was calculated as the sum of the hydropathy values of all amino acids divided by the number of residues in the sequence using the ProtParam (Expasy) program, which is available on the SIB Swiss Institute of Bioinformatics website.^15,16^ The secondary structures of HUG were predicted from the primary amino acid sequences of the polypeptide using GOR IV, the fourth version of the GOR secondary structure prediction methods, which uses all possible pair frequencies within the window of 17 amino acid residues. The simulation of the secondary structure of HUG using a multiple threading approach was performed on the I-TASSER server (Iterative Threading ASSEmbly Refinement) online platform.^17,18^

### 2.4 Turbidimetry

The transmittance of HUG samples at λ = 350 nm was measured in the range of *T* = 20–40 °C at a heating scan rate of 0.2 °C min^–1^ on a Jenway 6300 spectrophotometer. Transmittance data were converted to turbidity percent as (1 – *T*) × 100, and the turbidity reading was compared to a calibrated 100% transmittance reading of the filtered solvent as a blank. The inverse transition temperature (*T*_t_) was defined as the temperature corresponding to 50% of the maximum value. Purified polymers (HELP and HUG) were dissolved in phosphate buffer saline (PBS, pH = 7.4) or Tris buffer (pH = 8.0, 0.15 M NaCl) to a final concentration of 2 g L^–1^. Before measurements, the solutions were equilibrated at 4 °C for 16 h.

### 2.5 Differential Scanning Calorimetry

Thermal properties of HUG (lyophilized preparations) were evaluated using a Setaram Micro—DSC III. Differential scanning calorimetry (DSC) aluminum cells were filled by weight with protein samples (4 g L^–1^, in PBS, pH = 7.4), hermetically sealed, and equilibrated for 16 h at 4 °C. The calorimetric program consisted of a pre-equilibration phase at *T* = 5 °C for 10 min, followed by heating from 5 to 50 °C at a scan rate of 1 °C min^–1^. A solvent-containing cuvette was used as a reference. DSC measurements are always characterized by a broad peak extending over 20 °C or more. In this case, the inverse transition temperature (ITT) can be considered either the onset (*T*_ons_) or the peak temperature (*T*_p_, the maximum of heat absorption). The enthalpy (Δ*H*_tr_) and entropy (Δ*S*_tr_) of the transition were determined by area integration using in-house developed graphics software. Lysozyme was used as a calibration standard.

### 2.6 Circular Dichroism

HUG was dissolved in a concentration of 0.1 g L^–1^ in PBS pH = 7.4. Circular dichroism analysis (CD) was performed with the polymer alone and in the presence of the ligand BR. CD spectra were recorded at 25 °C in a thermostatic cell from 200 to 500 nm on a Jasco J-710 spectrometer under constant nitrogen purge. An external bath was used for temperature control. CD data are reported as the mean molar ellipticity [θ] of the residue in mdeg cm^2^ dmol^–1^.

### 2.7 Potentiometric Titration

Titrations were performed at room temperature. HUG (4 mg) was first dissolved in 2 mL of Milli-Q water. The aqueous solution in the form of the free acid was prepared by adding 2 g of Amberlite IR-120 Plus to 2 mL of the polymer solution. After stirring in an ice bath, the solution was filtered through a GF/F membrane. The pH values were measured using a HANNA pH meter with a glass microelectrode calibrated with standard buffers at pH = 4.01 and 7.00. The pH titration was performed by adding small volumes (3 μL in increments with a Hamilton precision syringe) of 0.1 N NaOH solution with stirring. The pH increase in the range of 2–11 was monitored as a function of the total volume of NaOH solution added. As the titration approached the equivalence point, small changes in the activity of the test substance solution triggered a dramatic change in pH. The fully protonated state and the fully deprotonated state (degree of protonation equal to 100 and 0%) were determined by the two extreme points of the first derivative of the pH titration curves.

### 2.8 Spectrophotometric Measurements of the BR Stock Solution

The BR stock solution was prepared from powder (#B4126; Sigma-Aldrich) by dissolving in dimethyl sulfoxide (DMSO) to 3 g L^–1^ (5 mM, stock solution). The BR working solution was prepared by diluting the stock solution to 10 μM BR in PBS containing 4 g L^–1^ BSA, pH = 7.4. Measurements of the intensity of the absorption spectra (ABS) of the 10 μM BR working solutions were performed using a dual-beam spectrophotometer (CARY-4E UV–visible spectrophotometer). Quartz cuvettes with a light path of 1 cm were used for the spectral measurements at room temperature between 350 and 600 nm.

### 2.9 Fluorometric Assay

All BR samples were prepared under subdued light and stored in brown bottles in a dark room until analysis because BR is light-sensitive and chemically labile. All experiments were performed with freshly prepared solutions at room temperature to avoid any degradation process. BR standard solutions ranging from 5 to 50 nM BR were prepared by diluting the 10 μM BR working solution in PBS with 0.4 g·L^–1^ BSA to obtain a standard curve.^19^ For each point, 200 μL of each BR standard solution was added to 10 μL of a HUG 1 g L^–1^ (PBS, pH = 7.4) in a 96-well microplate for fluorescence-based assays. The microplate was then incubated at room temperature for 2 h, and fluorescence emitted from the complex was detected at λ = 528 nm after excitation at λ = 485 nm using a benchtop microplate reader (Synergy H1; BioTek, Winooski, VT).

### 2.10 Dynamic and Static Laser Light Scattering

Laser light scattering measurements were performed using a Zetasizer Nano particle analyzer model ZS (Malvern Instruments). Dynamic light scattering (DLS) was performed using solutions of HUG and HELP at various temperatures and concentrations (*C* = 0.1–0.5 g L^–1^). Scattering intensities were measured at an angle of 173° (backscattering) with an incident laser wavelength of 633 nm (size diameter range 0.3 nm to 10 μm). The percentage of the peak areas was obtained from intensities, and size is an intensity-based calculated value. The intensity distribution is weighted according to the scattering intensity of each particle fraction.

The relationship between the size of a particle and its velocity due to Brownian motion is defined in Stokes–Einstein theory. The intensity, volume, and number distributions can be calculated by fitting the autocorrelation function measured in the experiment. This analysis implies a nonlinear least squares fitting (NLLS) and smoothing parameter. The particle size distribution from DLS is derived by deconvolution of the experimental intensity autocorrelation function of the samples. This is obtained using a non-negative constrained least squares (NNLS) fitting algorithm such as CONTIN. Multiexponential fitting is more appropriate and is used here for broader and multimodal distributions. The diffusion coefficients *D* obtained from the fitted data were used to calculate the mean hydrodynamic radii *R*_h_ using the Stokes–Einstein equation^31^
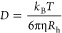
where *k*_B_ is the Boltzmann constant, *T* is the temperature, and η is the viscosity of the solvent.

Static light scattering (SLS) was performed on dilute protein solutions (starting solution *C* = 0.1 g L^–1^). The intensities measured at an angle of 7° were used to calculate the scattering ratio and plotted as a Debye plot (scattering ratio *vs* concentration). The weight-average molecular weight (*M*_w_) and second virial coefficient (*A*_2_) were determined by linear fitting of the Debye plot, as reported in Figure S4. Toluene was used as the calibration solvent.

## 3 Results

### 3.1 Predicted Macromolecular Features of HUG

A preliminary analysis of HUG chain features in relation to HELP and its secondary structure was performed by simulating biopolymer properties using the Expasy platform. The physicochemical parameters of HUG (Table 1) were calculated from its primary structure and compared with those of HELP and UnaG.^6,20^

**Table 1:** Physicochemical Parameters of HUG and Related Proteins^a^

	MW	p.I.	hydropathy index (GRAVY)	polar a.a. (%)	charged a.a. (%)	aromaticity (as Tyr + Trp + Phe) (%)
HUG	60,406	9.9	0.77	5.3	10.0	3.1
HELP	44,886	11.7	1.1	1.9	3.2	1.5
UnaG^b^	15,581	6.61	–0.49	20.9	32.3	9.3

a Data were obtained using Expasy Tools (ProtParam on-line software)

b UnaG DDBJ/EMBL/GenBank databases (accession number AB763906).

The content of charged and polar amino acids (15.3%) of the biopolymer HUG (calculated *M*_W_ = 60,406 Da) is greater than that of HELP (5.1%). The presence of the UnaG domain in HUG leads to a significant difference in the hydropathy index (Table 1) compared to the HELP biopolymer. This could have implications for the increased solubility of the HUG biopolymer, but this property needs further experimental investigation.

Table 2 shows the results in terms of secondary structure distribution for the HUG biopolymer compared to the results for HELP and UnaG. As highlighted in a previous article, the distribution of the secondary structure of HELP is very similar to human elastin with a prevalence of disordered coil and β-turn domains (60–70%).^20^ Considering the predicted HUG secondary structure, the UnaG domain (large β-strand present, 35%) fused at the C-terminus of the HELP sequence introduces into the HUG chain 49 a.a. (35% of 139 a.a), which have the β-strand conformation.

**Table 2:** Prediction of the Secondary Structure of HUG and Related Proteins^a^

	number of a.a.	α-helix %	β-strand %	random coil + β-turn %
HUG	675	22	10	68
HELP	536	26	4	70
UnaG^b^	139	8	35	57

a Data were obtained using GOR IV.

b UnaG DDBJ/EMBL/GenBank databases (accession number AB763906).

Given these preliminary results, an in-depth simulation of the secondary structure of the polymer HUG was performed. For this purpose, the online platform I-TASSER server was used.^17,18^ Protein structure and function prediction by I-TASSER algorithms is a hierarchical approach that enables the generation of high-quality 3D model predictions and biological functions of proteins starting from the primary structure through a multiple threading method.

**Figure 1:**
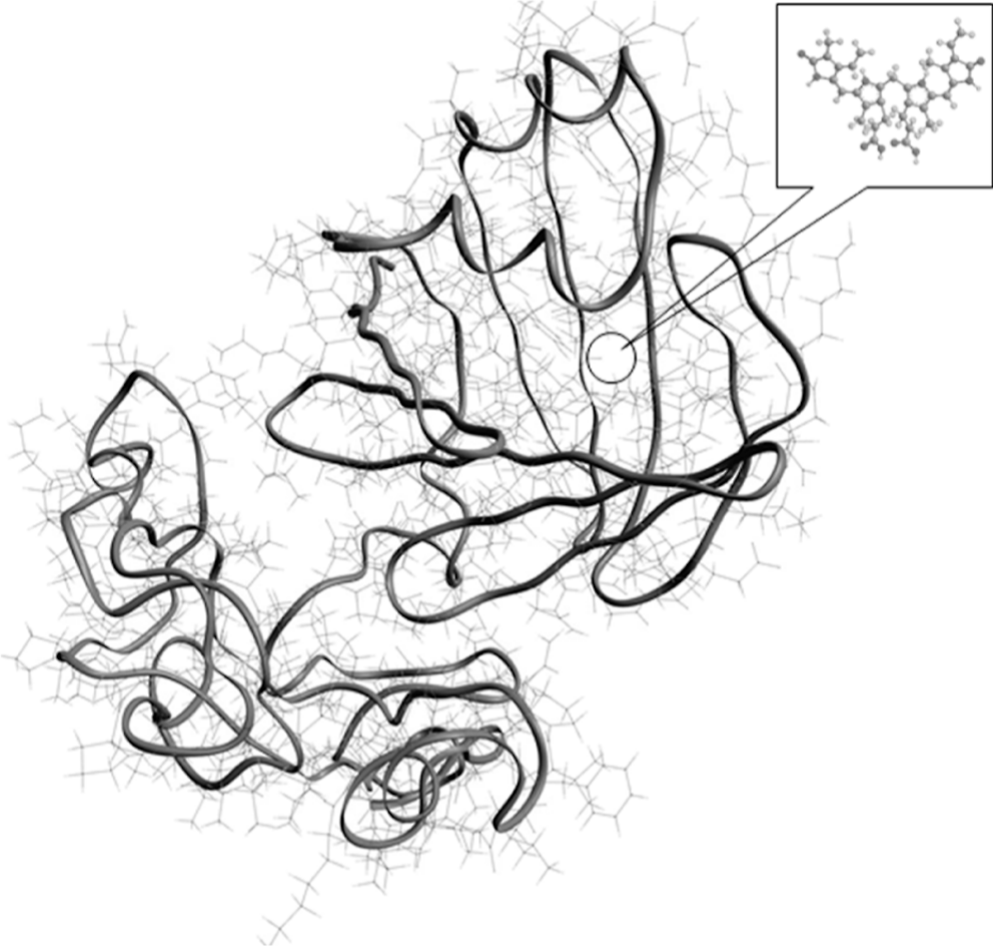
Model of the secondary structure of HUG. The model is a I-TASSER minimized structure of HUG, with the UnaG domain connected to a fragment of the HELP biopolymer.

**Figure 2:**
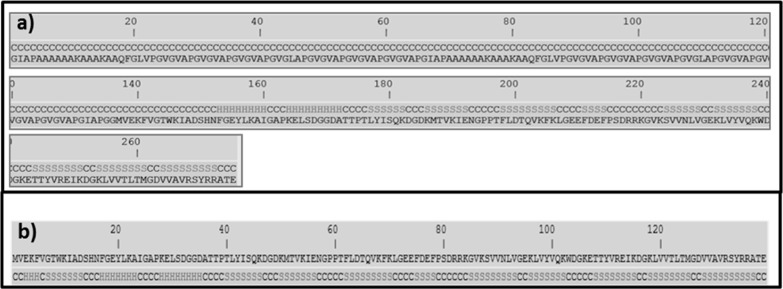
Prediction of the secondary structure of a portion of HUG and of UnaG. (a) Primary structure of HUG starting from a.a. 400 and (b) primary structure of UnaG. Both are associated to the secondary structures (C = coil, H = α-helix, S = β-strand), obtained by I-TASSER simulation.

A snapshot of a minimized structure resulting from the calculations of I-TASSER for one region of the protein HUG (275 out of a total of 675 HUG a.a.) is shown in Figure 1, where the two different regions of the protein are shown, that is, part of the domain HELP of 136 a.a. (out of 400 to 536 residues of the biopolymer HELP, Figure 2a) and the entire UnaG domain (139 a.a Figure 2b). Interestingly, simulation showed that both sequences, HELP and UnaG, retain their own secondary structures, even in the HUG fusion protein, as shown in Figure 2. The HELP region adopts the coil conformation (100%), while the UnaG domain shows the β-strand (46%), coil conformation (41%), and helix conformation (12%), which according to Kumagai and co-workers is the same as that of UnaG itself.^5^ These results are consistent with those in Table 2, which were obtained using the GRAVY method.

The 3D model in Figure 1 shows the structural basis for confirming that the HUG fusion protein retains the major macromolecular features of each domain. Evaluation of the biophysical properties of HUG in solution was performed to determine the most appropriate conditions for using the properties of this fusion protein for bilirubin analysis.

### 3.2 Physicochemical Properties of HUG in Solution

The bifunctional properties of HUG were investigated using different approaches to reveal possible differences with respect to HELP and the UnaG properties reported in the literature.^1,5,21^

#### 3.2.1 HUG Secondary Structure Analysis

**Figure 3:**
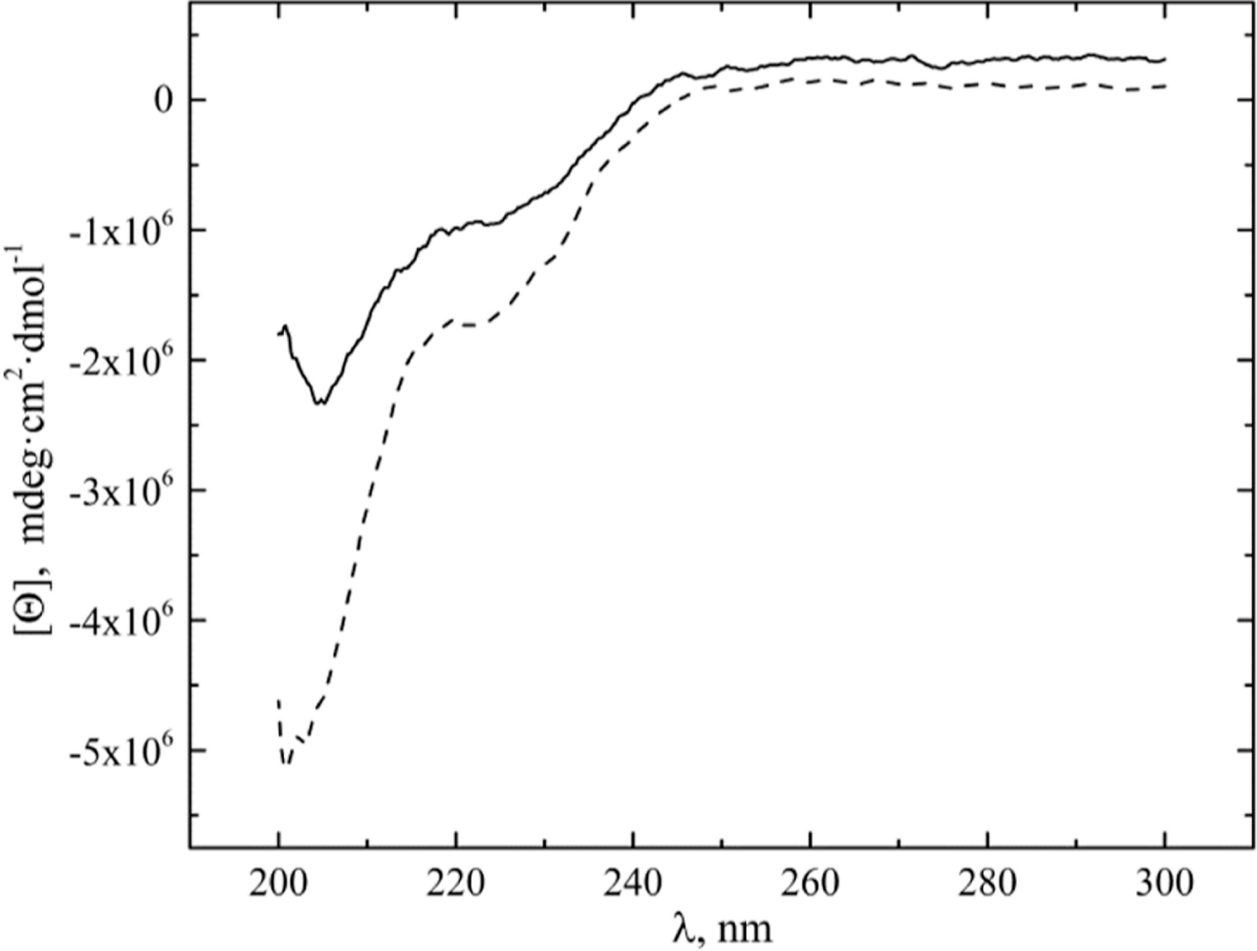
CD spectroscopy of HUG and HELP. HUG (^___^) and HELP (---) polymers were solved in PBS solution, *C* = 0.1 mg·mL^–1^, pH = 7.4, *T* = 25 °C.

CD spectra obtained at a temperature below the ITT (*T* = 25 °C) for the HUG and the HELP biopolymers (Figure 3) show the typical profile reported for the elastin-like polypeptides, with a negative band around 200 nm (ππ*—transition), associated with the coexistence of different conformer populations varying between a high proportion of random coils and a low proportion of β-turn and PP-II secondary structures with very similar conformational parameters.^22,23^ The difference in CD signal at 200 nm between HUG and HELP is due to the large positive contribution of the β-structure at this wavelength attributed to the HUG domain.

The presence of additional negative bands and a less pronounced minimum at 222 nm (nπ*—transition) at longer wavelengths has been reported previously and attributed to both α-helical domains (222 nm) and type I/type II β-turn conformations (225 nm).^20^

The CD spectra of HELP and HUG remained unchanged when different concentrations of BR were added to both biopolymer solutions (Figure S2 in the Supporting Information), consistent with data reported by other authors for UnaG.^21^

#### 3.2.2 Polyelectrolyte Features

Potentiometric titrations can provide information about possible pH-induced hydrophobic folding and assembly transitions.

**Figure 4:**
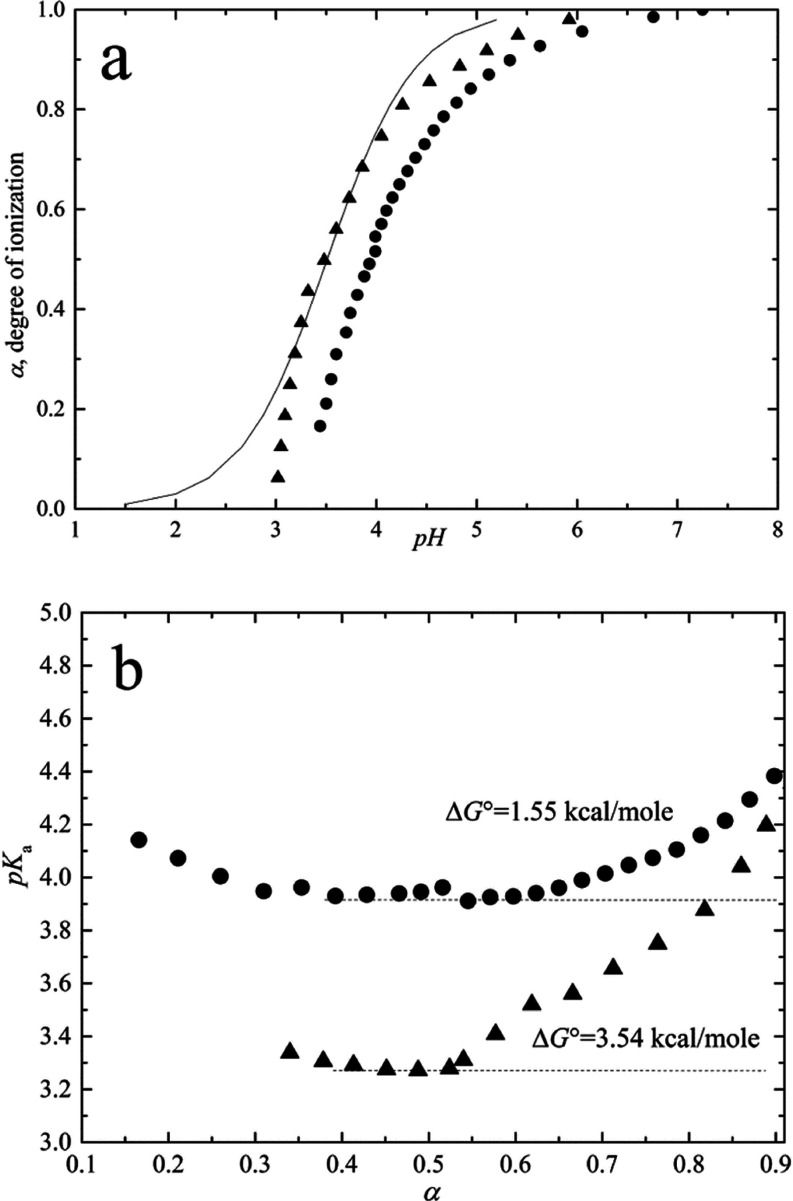
Potentiometric analysis of HUG and HELP. (a) Titration curves for acid-dialyzed proteins HUG (triangle) and HELP (circles). The solid line represents the Henderson–Hasselbalch equation. (b) Dependence of p*K*_a_ on the degree of protonation (α).

Figure 4a shows the data giving the sigmoid curve for the titration of the acidic moiety in terms of the degree of ionization, α, as a function of pH. Figure 4a also shows the theoretical (solid) curve for the Henderson–Hasselbalch equation
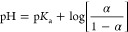


The experimental curve is clearly much steeper than the theoretical curve for a weak acid showing a positive cooperativity. In the equation, α is the mole fraction of the titrated functional group (the degree of ionization)
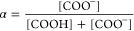


*K*_a_ is the unperturbed value for the equilibrium constant between two states (COOH and COO^–^) in relation with the Gibbs free energy as
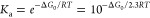


Significant deviations from the Henderson–Hasselbalch equation have been reported in the literature for polypeptides containing acidic amino acids and having several intervening hydrophobic residues.^24,25^ It has been extensively discussed and experimentally demonstrated that a progressive increase in hydrophobicity causes a progressive increase in the slope (positive cooperativity) of the titration curve and a shift in p*K*_a_.^24,26^ The increase in p*K*_a_ is a consequence of the formation of charged species during titration, which is responsible for the de-structuring of the water molecules in the pentagonal arrangement of the hydrophobic hydration structure. In other words, the newly formed COO^–^ groups pull water from the hydrophobic hydration shells into their own hydration shells. The increase in charge density during titration causes an increasing charge–charge repulsion effect, which increases the free energy of the system and a continuous change in p*K* value (negative cooperativity) for α > 0.3 (Figure 4b).^27^

In the presence of charge–charge repulsion, the slope of the titration curve is much sharper than that given by the Henderson–Hasselbalch equation. The deviation from the Henderson–Hasselbalch equation is usually evaluated by introduction of the Hill coefficient ***n***
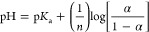
where ***n*** introduces the cooperativity into the acid–base titration theory and p*K*_a_ = p*K*_0_ + Δp*K* due to the cooperative actions of acidic residues and to the significant hydrophobic hydration. The Hill coefficient and the Δp*K* = ΔΔ*G*_0_/2.3*RT* could be estimated by the Wyman equation of free energy^28^
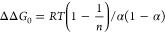
where for α = 0.5
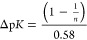


Considering the theoretical models for weak polyelectrolytes of Harris and Rice and of Katchalsky and Gillis, the last term of the above pH equation can be expressed as^29,30^
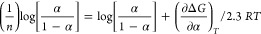


The last term describes the steepness of the titration curves compared to that obtained with the equation of Henderson–Hasselbalch as
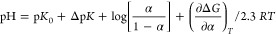


Moreover, the free-energy contribution ∂Δ*G*/∂α takes accounts for the two mechanisms responsible for the p*K* shifts and the steepness of titration curves, that is, the charge–charge repulsion (c–c) and the apolar–polar (a–p) repulsive hydration free energies and related constants
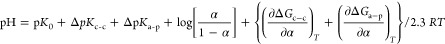


The first partial derivative (c–c) refers to a *negative cooperativity* describing a titration curve broader than that obtained by the equation of Henderson–Hasselbalch, while the second derivative (a–p) refers to a *positive cooperativity* with a steeper sigmoidal curve. Therefore, a competition for hydration water molecules exists between hydrophobic and charged domains.

The increase in Δp*K* due to charge–charge repulsion (Figure 4b) broadens the titration curve (negative cooperativity) of both HUG and HELP protein in solution at a temperature below the inverse *T*_t_ so that they remain in solution throughout the titration. Under these conditions, both proteins show no hydrophobic folding and assembly transition so that the Δp*K* shift is mainly due to the work required to disrupt the hydrophobic hydration structure.

By integrating the Δp*K* curves, it is possible to derive the change in free energy Δ*G* associated with the charging process during titration
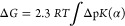


At *T* = 22 °C, *RT* = 0.584 kcal·mol^–1^, so Δ*G* is equal to 1.55 and 3.54 kcal·mol^–1^ for HELP and HUG biopolymers, respectively (Figure 4b), given the higher charged residue content of HUG (10%) compared to the HELP biopolymer (3.2%) (Table 1).

#### 3.2.3 Inverse Thermal Transition

**Figure 5:**
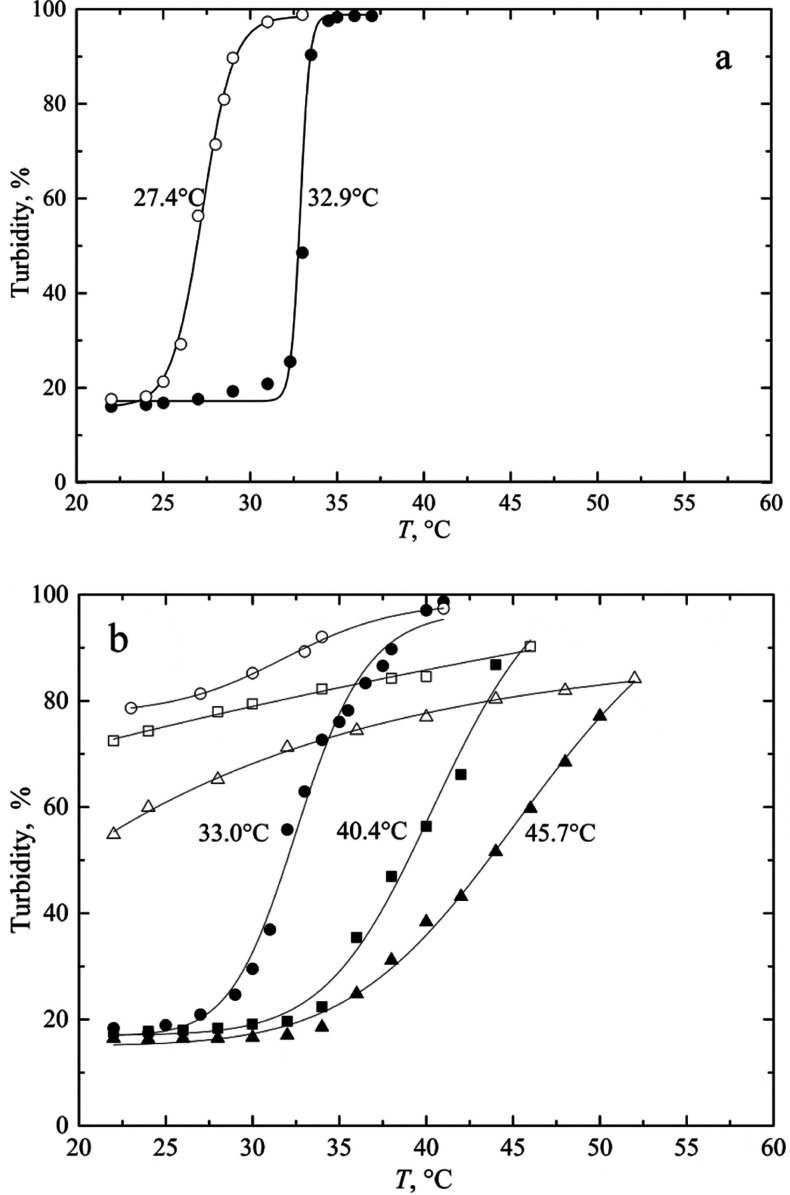
Turbidimetric analysis of HELP and HUG solutions as a function of temperature. Solutions in PBS, pH = 7.4) of HELP (a, *C* = 2 g·L^–1^) and HUG (b) were heated (solid symbols) up to steady-state turbidity and then cooled down (open symbols). HUG concentrations: (triangles) 0.5 g L^–1^, (squares) 1 g L^–1^, (circles) 2.0 g L^–1^.

Turbidimetric and calorimetric measurements were performed to follow the ITT of the protein in solution. The transition temperature (*T*_t_) is a suitable parameter to describe the tendency of elastin-like biopolymers to undergo hydrophobic folding and the transition known as coacervation.^24^ The turbidity profile shows a sharp increase near the thermal transition, and the corresponding temperature is that corresponding to a 50% change in the relative turbidity of the solution (Figure 5). Both biopolymers show a complete transition occurring in a narrow range of 2–3 °C as the temperature increases from 20 to 50 °C.

For the protein HELP, complete reversibility with net hysteresis of the process was observed upon cooling (Figure 5a), whereas the solution HUG did not re-dissolve completely upon cooling under these conditions and did not show similar structural recovery (Figure 5b). The cooling process in Figure 5b shows a time-dependent behavior that is not studied in detail in this paper. In a short time interval, irreversibility of the polymer HUG was observed, while at longer times, complete dissolution occurred. Comparing these results with those previously obtained for the biopolymer HELP, the data summarized in Table 3 for different solvent conditions show a very small shift in the transition temperature *T*_t_ (at 50% of transition) of HUG and HELP for *C* = 2 g L^–1^ with respect to the primary structural differences between the two biopolymers.^20^ The dependence of ITT on polymer concentration of HUG is also given in Table 3 with an increase in *T*_t_ from 33.0 °C to 45.7 °C in PBS solution at pH = 4, going from 2 to 0.5 g·L^–1^.

**Table 3:** Dependence of Transition Temperature on Solvent Properties and Polymer Concentration for HELP and HUG Assessed by Turbidimetric Analysis During Heating

	HELP		HUG	
solvent	*C*, g·L^–1^	*T*_t_, °C	*C*, g·L^–1^	*T*_t_, °C
Tris buffer pH = 8, 0.15 M NaCl	2.0	32.7	2.0	31.9
PBS pH = 7.4		32.9	2.0	33.0
			1.0	40.4
			0.5	45.7

**Figure 6:**
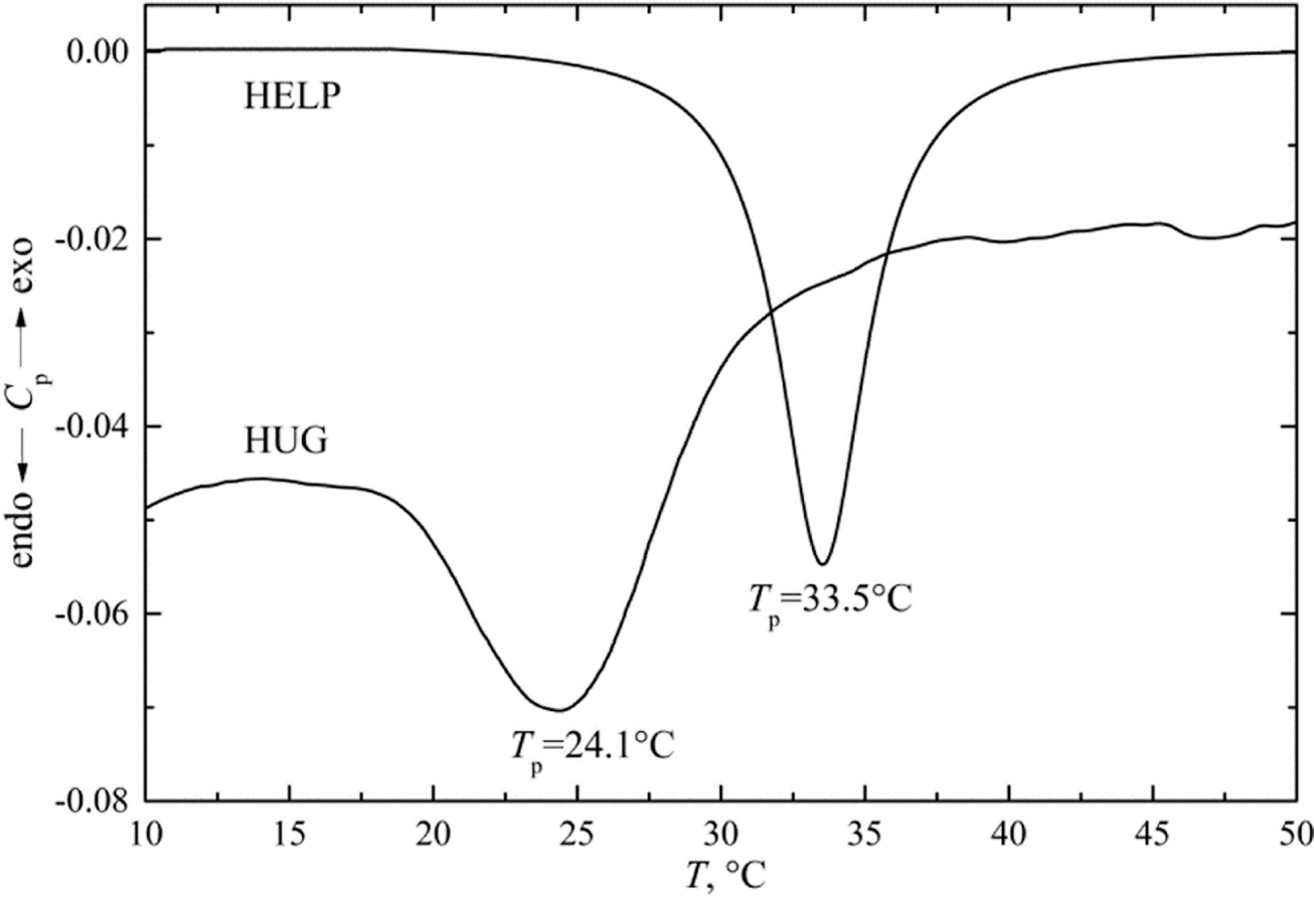
DSC thermograms of HELP and HUG. Solutions (4 g L^–1^, in PBS, pH = 7.4) were analyzed at scan rate of 0.5 °C min^–1^.

DSC measurements of ITT are always characterized by a broad peak extending around 10 °C or more for both biopolymers. The ITT can be considered as either the initial or peak temperature. Figure 6 shows representative heating DSC thermograms for both biopolymers HUG and HELP.

Under these experimental conditions, the peak and onset values (*T*_p_, *T*_ons_) of the inverse transition temperatures and thermodynamic properties (Δ*H*_tr_ and Δ*S*_tr_) during heating are listed in Table 4. The transition enthalpy and entropy, Δ*H*_tr_ and Δ*S*_tr_, were determined by integrating the *C*_p_ and *C*_p_/*T* data from the DSC experiments, respectively.

**Table 4:** Onset and Peak Temperature, Enthalpy, and Entropy Variation for HUG and HELP Proteins From DSC Measurements (HUG and HELP 4 g L^–1^ in PBS, pH = 7.4)

	*T*_ons_, °C	*T*_p_, °C	Δ*H*_tr_, kJ·mol^–1^	Δ*S*_tr_, kJ·mol^–1^K^–1^
HUG	19.0	24.1	158	12.4
HELP	29.4	33.5	216	12.8

These data showed a significant difference in temperatures and enthalpies between HUG and HELP biopolymers, mainly due to the different contribution of structural water order disruption associated with the extent of chain hydrophobicity, which is higher for HELP protein than for HUG. Moreover, the entropy change during the inverse transition is similar, suggesting that the very extended hydrophobic spheres of solvation of these two biopolymers have a large number of water molecules that contribute similarly to the transition entropy. The significant difference in Δ*H*_tr_ values in Table 4 can probably be attributed to the charged groups, which account for more than 7% in HUG compared to the HELP biopolymer (Table 1). Finally, a difference between the ITT values obtained with DSC and turbidimetry can be observed, especially for HUG. Because the concentrations for the turbidity experiments were in the range of about 2 g L^–1^, whereas the concentrations for DSC were generally in the range of 4–10 g L^–1^, the differences in *T*_t_ could be related to the concentration effect on protein folding and to the cooperativity of the process. Moreover, *T*_t_ values obtained by these methods differ due to the dynamic nature of DSC and its relative thermal lag, which is higher at higher heating rates. This indication could be related to the different thermodynamic or optical properties observed and due to the concentration effects and to the heating rate.

### 3.6 Particle Dimensions from Light Scattering Measurements

Dynamic light scattering (DLS) measures Brownian motion and relates it to particle size by illuminating particles with a laser and analyzing the intensity fluctuations in the scattered light.^31^

**Figure 7:**
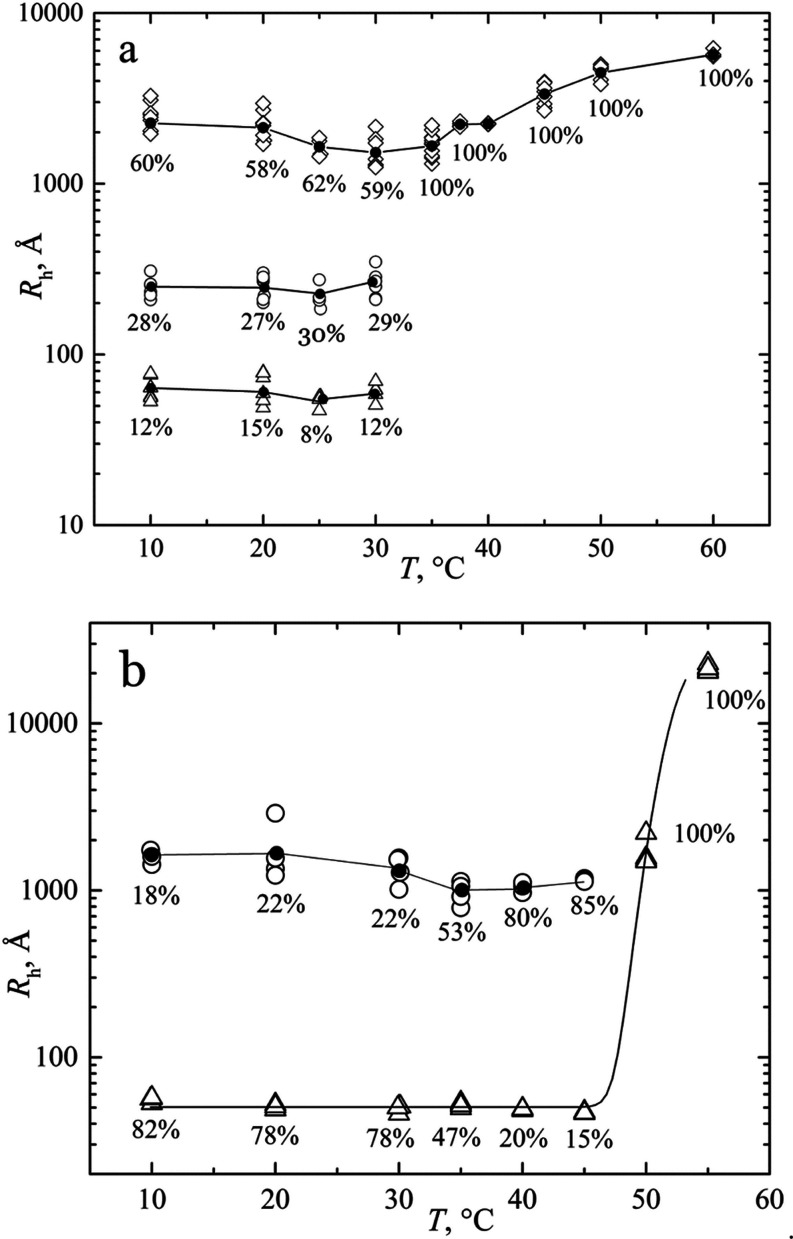
Temperature dependence of the hydrodynamic radii of HUG and HELP. (a) HUG values: (Δ) *R*_h_ = 62 Å, (○) *R*_h_ = 250 Å, (◊) *R*_h_ = 1523–2497 Å. (b) HELP average values: (Δ) *R*_h_ = 50 Å, (○) *R*_h_ = 1350 Å. Filled circles are average values.

Using the DLS technique, therefore, we were able to measure the hydrodynamic radius of macromolecules in solution and dimensions of aggregates of different sizes at different temperatures and concentrations. Typical results are shown in Figure 7a for the biopolymer HUG and in Figure 7b for HELP to compare the behavior of the two proteins. The radius *R*_h_ for HUG was measured as a function of *T* from 10 to 60 °C to evaluate the size distribution of particles during heating; the results are shown in Figure 7a.

Three modal size distributions were observed for the protein HUG in the temperature range of 10–30 °C with an average *R*_h_ value of 62, 250, and 1523–2497 Å, respectively (Figure 7a). The typical correlation function and size distribution are shown in Figure S6 of the Supporting Information. These three distributions differed with respect to the percentage of peak areas, indicating that the particles with the highest size in this solution condition are the main fraction up to 30 °C (Figure 7a). As the temperature of HUG increases, the protein undergoes the inverse phase transition and the peaks resolve into a single modal distribution, forming huge complex aggregates with increasingly very large hydrodynamic radii up to 6000 Å. It is noticeable that *R*_h_ for the sample HUG has its minimum value at 25–30 °C; then the diameter of the aggregates increases and the reverse transition occurs. This observation could be useful if the HUG was dissolved and suggests that a room-temperature step should be provided before cooling the solution. Coacervation coincided with a percent intensity distribution shift from a widely distributed population of particles below ITT to a single peak above 30 °C. The particle size distribution remains single modal above the coacervation temperature.

This behavior was similar to that observed for the biopolymer HELP (Figure 7b and Figure S5 in the Supporting Information). In this case, the most important polypeptide fraction in the temperature range of 10–30 °C was the one with about *R*_h_ = 50 Å (78%) together with that of about *R*_h_ = 350 Å (22%) (Figure 7b). By increasing the temperature, the protein showed an initial aggregation forming particles with an average *R*_h_ value greater than 1000 Å. Complete coacervation was then achieved at *T* > 50 °C where a single modal distribution was observed.

**Figure 8:**
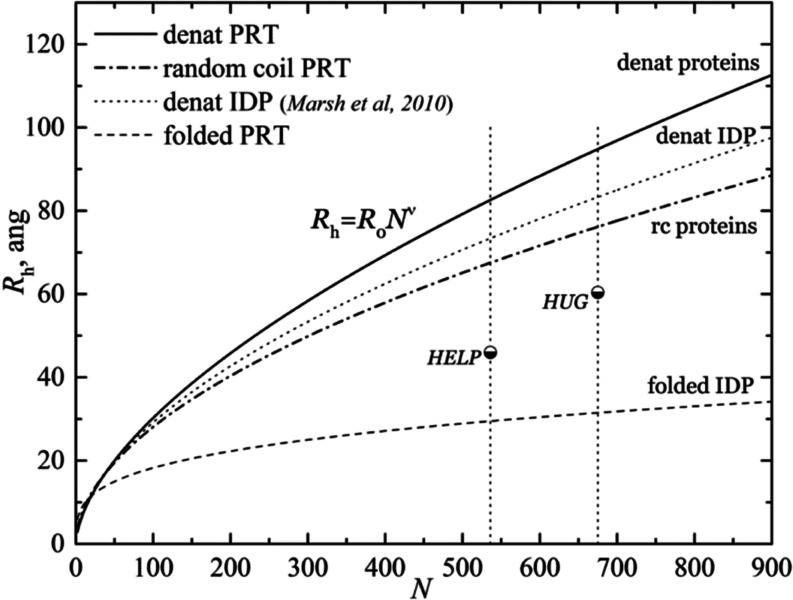
Hydrodynamic radii of HUG and HELP particles in comparison with other classes of proteins. The plot shows the dependence of the hydrodynamic radius (*R*_h_) of a protein on the number (*N*) of amino acids in its primary structure. Further details are in the text.

DLS data of the solutions of HUG show that a single population of very large particles with a diameter of 2000–6000 Å became evident after coacervation when the temperature was increased above 35 °C. At *T* < 35 °C, HUG is present in a fraction of 10–15% as a single chain of about *R*_h_ = 60 Å, which is higher than that of the HELP polymer (average *R*_h_ = 46 Å) as shown in Figure 8. The same figure also shows *R*_h_ curves obtained by fitting a large number of proteins (random coil, folded, denatured, and IDP proteins) as a function of the number of a.a. residues, *N*.^32,33^

It is known from Flory’s work in the mid-20th century that polymers show a power-law dependence, where the radius of gyration is proportional to the number of residues raised to a power.^34^ Each protein type set was fitted with a power-law scaling equation
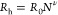
where *R*_0_ and ν are constants obtained by fitting of the huge protein data set and are listed in Table 5.^32^

**Table 5:** Parameters of the Power-Law Scaling Equation From Marsh and Co-workers^32^

	*R*_0_, Å	ν
denatured proteins	1.927	0.598
random coil proteins	2.54	0.522
denatured IDPs	2.33	0.549
folded proteins	4.92	0.285

The exponent ν is considered as a universal constant and interpreted as a measure of the compactness of the polymeric chain. The *R*_h_ curve for random coils shown in Figure 8 can be considered as a defining separation between folded and IDP proteins. The folded proteins were all below the random coil line, and the denatured IDPs were all more frequently above this line.^32,35^ As observed by Urry and co-workers for elastin-like proteins and by our CD results, HELP and HUG chains not only preferentially adopt a random coil conformation but also are partially composed of beta-turns that exhibit a compact, partially folded globular conformation due to preferential chain–chain interactions, as observed in Figure 8.^36^

Molecular weight measurements were performed using a static light scattering procedure (SLS). Instead of measuring the time-dependent variations in scattered light intensity, SLS uses the time-averaged intensity of the scattered light at an angle around 0 (7°). The intensity of the scattered light over a period of time (*e.g.*, 10–30 s) is accumulated for a range of concentrations of the polymer sample. SLS measures the intensity of scattered light (expressed as the ratio *KC*/*R*_θ_ in the Debye equation below) of different concentrations (*C*) of the sample at an angle, where *K* is the scattering vector. *R*_θ_ is the Rayleigh ratio of the polymer solution and is calculated using the toluene Rayleigh ratio, *R*_T_, which can be found as a general standard in many reference works. Using the Debye equation, we can determine the molecular weight (*M*_w_) and second virial coefficient (*A*_2_) of the polymer in a given solvent medium from the equation
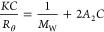


The weight-average molecular weight (*M*_w_) is determined from the intercept at concentration *C* = 0, that is, *KC*/*R*_θ_ = 1/*M*_w_ (for *c* → 0), where the *M*_w_ is expressed in kDa. The *A*_2_ coefficient is determined from the gradient of the Debye plot. The static light scattering results, that is, the averaged *M*_w_ and *A*_2_, for HUG and HELP diluted protein solutions are shown in Table 6. Figure S4 in the Supporting Information shows one of the representative Debye scatter plots used for the average calculations. The experimentally determined *M*_w_ data agree well with the *M*_w_ calculated on the basis of the primary structure of both proteins. These results confirm that the polymers expressed by the bacteria correspond to the primary sequence deduced from the gene sequences and that no degradation of the proteins occurred during the purification process.

**Table 6:** Mean Static Light Scattering Results (*n* = 4) at 25 °C in 0.15 M NaCl Solution From Debye Plots

	theoretical *M*_w_, kDa	*M*_w_, kDa	*A*_2_, mL mol g^–2^
HUG	60.4	65.3 ± 5.7	–0.049 ± 0.024
HELP	44.9	40.5 ± 0.46	–0.065 ± 0.053

The second virial coefficient *A*_2_ was always negative, indicating a weak interaction between polymer chains and solvent molecules (poor solvent), which is why these polymers tend to aggregate.

### 3.7 BR Binding to HUG Protein

**Figure 9:**
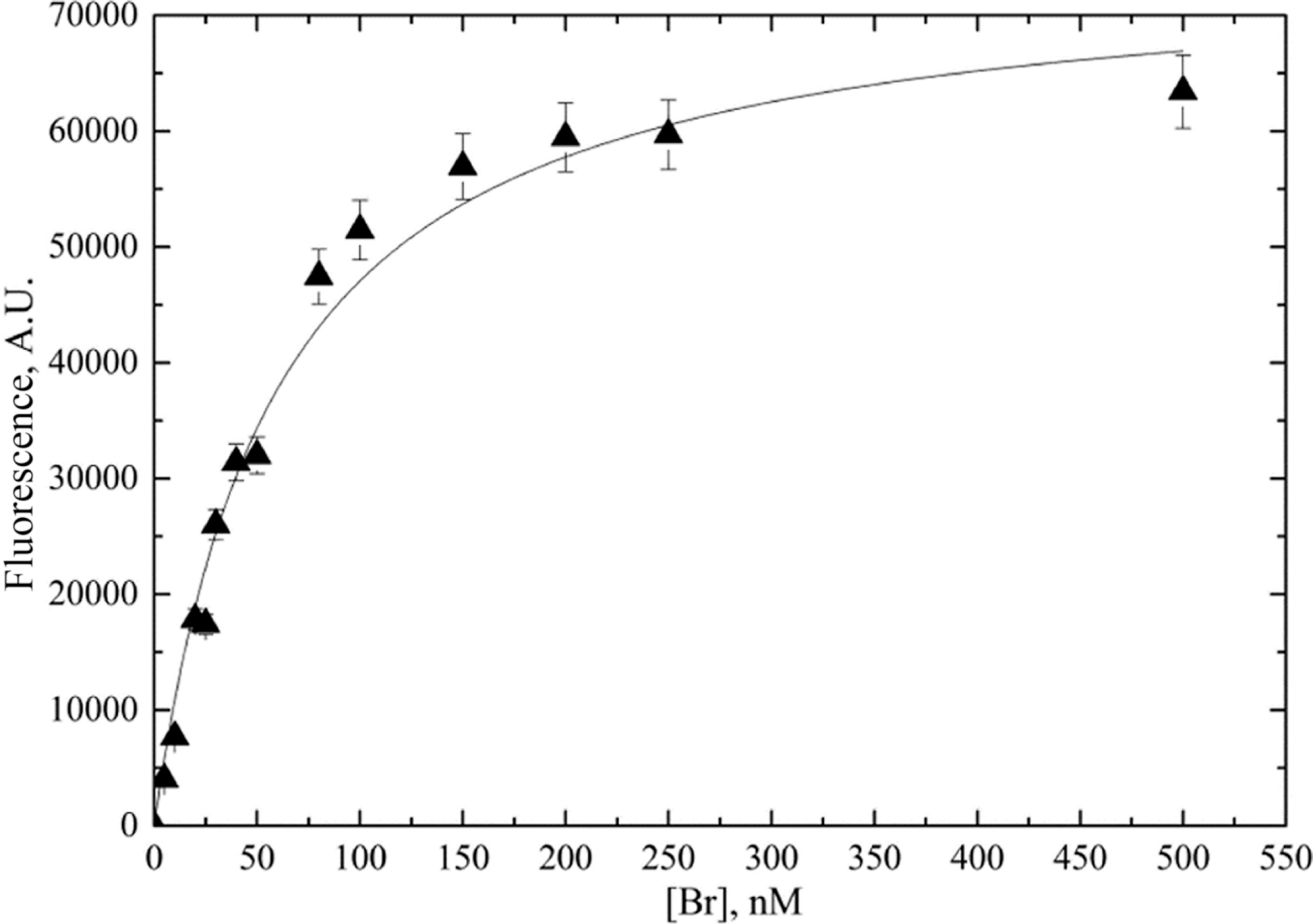
HUG titration with BR. HUG (140 μM) was incubated with a series of BR concentrations in the presence of BSA (0.4 g L^–1^) at 25 °C for 2 h.

The BR-HUG interaction was studied using the fluorescence titration technique, which evaluates the increase in intensity during the addition of the ligand.^37,38^ Titration was performed by adding a BR solution ranging from 0 to 500 nM at 25 °C to a constant amount of HUG (*C* = 0.140 μM), and the results are reported in Figure 9.

For each fluorescence intensity value (*F*), the fractional enhancement (*Y*) was computed by the equation
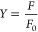
where *Y* is the fractional saturation related to the extent of binding and *F*_0_ is the fluorescence intensity at the BR/HUG ratio greater than 1 (the asymptotic value). Given the simple case of single binding of small molecules to independent identical sites on a macromolecule, the chemical expression describing the binding process is^39,40^
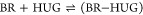


The equilibrium dissociation constant, *K*_d_ = 1/*K*_a_, where *K*_a_ is the association constant, is a measure of the strength of the interaction, that is,
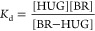


During the binding titration, the BR concentration is increased so that saturation *Y* is expressed in terms of the BR–HUG complex concentration, [BR–HUG], as
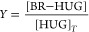
1where [HUG]_*T*_ is the total HUG concentration used for the measurement. Then
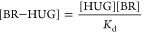

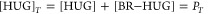

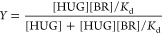

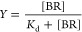


Since in the binding measurement instead of free concentrations the total protein and ligand concentrations are known, an expression of *Y* as a function of total quantities is derived as follows
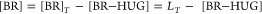
then
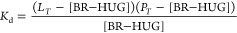

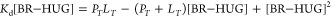

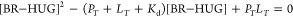


The root of the equation is
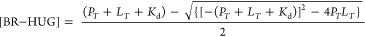


By substitution in eq 1
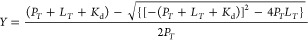
2

The plot of *F* = *F*_0_*Y versus L*_*T*_ = [BR] is shown in Figure 9, where the average values of all the results obtained by several experiments are reported. Equation 2 can be used directly to analyze data from titration experiments of *F vs* [BR] in the 1:1 binding model. By nonlinear least-squares fitting of the hyperbolic curve, the best evaluation of equilibrium dissociation constant, *K*_d_ = 1.1 × 10^9^ M, or expressed as binding constant *K*_a_ = 1/*K*_d_ = 0.91 × 10^9^ M^–1^, and of the maximal fluorescence value of the ligand-bound protein (*F*_0_ = 65,568) was obtained. The assumption in the model of unitary binding capacity was confirmed from the Scatchard plot (*n* = 1.1) (Figure S3 in the Supporting Information) following the Levine method.^40^

The value of the binding constant *K*_a_ = 0.91 × 10^9^ M^–1^ for the BR–HUG complex we measured gave a larger value than the *K*_a_ ≈ 10^7^ value for the albumin-BR binding, leading to the displacement of BR from albumin by competitive binding of HUG.^41,42^

## 4 Discussion

As described in previous articles, many studies have been published on the structure–property relationship of elastin-like proteins.^24,43−45^ However, there is still little evidence on the extent to which protein adducts can affect the properties of elastin-like proteins in solution. The fusion protein HUG is therefore an excellent model to test whether the typical properties of HELP are perturbed or preserved by fusion with UnaG.

In the first part of this work, an in silico analysis with the free software Expasy allowed the calculation of the hydropathy value (GRAVY) of the HUG polypeptide from its complete amino acid sequence.^16^ The calculated secondary structures of the UnaG domain (mainly β-strand conformation) and the HELP segment (100% coil conformation) at temperatures below the onset of the inverse temperature transition are typical of noncoacerved polymers and are consistent with previous CD and Raman spectroscopy observations of other proteins containing a large amount of short and irregular β-segments.^46,47^ In this study, most of these predictions were confirmed in spectroscopy, potentiometry, and light scattering studies of both HUG and HELP solutions.

The CD spectra of HUG showed a broad negative band at 200 nm, supporting the view that the protein is mainly disordered, although the overall shape of the spectra could be assigned to short and distorted β-sheets.^48^ The hexapeptide VAPGVG motif, present in both HUG and HELP, contributes strongly to their secondary structures, resulting in similar CD spectra as previously reported.^20^

The tertiary structure of the polymer HUG was investigated by following the transition of folding and assembly that occurs when the temperature is increased above a critical point. Above this threshold temperature, the reverse transition occurs as the intramolecular hydrophobic contacts between the VAPGVG domains are optimized. The results of turbidimetric and DSC analyses showed that HUG tends to undergo an ITT similar to HELP, with distinct threshold transition temperatures and thermodynamic properties at the selected salt concentration and pH condition (Tables 3 and 4). As previously reported, a lower hydrophobicity index (or a higher fraction of polar and charged residues) is associated with a lower transition enthalpy.^20,49^

DSC experiments have shown that the endothermic transition enthalpy depends significantly on the number of water molecules of the hydrophobic hydration to be destructured. Thus, the decrease in *T*_t_ of HUG compared to HELP from 33.5 to 24.1 °C is related to the lower hydrophobicity of the HUG moiety. DSC analysis and turbidimetric analysis revealed significant differences in the initial transition temperature, that is, *T*_ons_ = 19.0 °C for HUG and *T*_ons_ = 29.4 °C for HELP protein and *T*_ons_ = 30.7 °C and *T*_ons_ = 32.4 °C, respectively (Tables 3 and 4).

The ultimate supramolecular structure of HUG is gradually reached above the threshold for the reverse temperature transition, which is due to the collapse and aggregation of peptide molecules. When the temperature is increased for proteins with multiple β-strands, they combine through intermolecular hydrophobic interactions to form a coacerved system.^50^ At a given temperature within the inverse temperature transition interval, the polymer in water is in conformational equilibrium such that Δ*G* = 0, and the following relationships hold for the inverse temperature transition
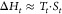


Since ITT is an endothermic transition, Δ*H*_t_ is positive, so Δ*S*_t_ is also positive. During the transition, the protein is more restricted in its movement by hydrophobic aggregations, leading to the formation of filaments and fibrils, as shown/discussed for other similar proteins.^51,52^ In this two-component system, the entropy increases when phase separation occurs. This means that even if the protein becomes more ordered, that is, the Δ*S*_t_ of the protein is negative, the water molecules contribute significantly to the positive Δ*S*_t_ of the system by becoming less ordered as bulk water and represent the positive entropy change that drives the ITT process (Table 4).

The thermal behavior and conformational change of HUG were also studied using light scattering techniques (DLS and SLS) to obtain information about the size and shape of this protein, that is, its hydrodynamic properties. In Figure 1, the minimized conformation of the HUG fragment shows an asymmetric shape that cannot be assimilated with either a compact globular secondary structure or a fully extended secondary structure. The case of partially ordered, partially flexible, and partially unfolded biomacromolecules has become a new area of interest in recent years, termed intrinsically disordered proteins (IDPs).^53,54^ These proteins exhibit interesting structural features that differ from the secondary structures of random coils normally found in denatured proteins. Hexapeptidic VAPGVG motif-based HUG and HELP are fully disordered and compactly folded polypeptides composed only of these peptide motifs with a net charge of zero and moderate hydrophobicity.^20,55^ One aspect that HELP and its fusion derivatives have in common with IDPs is the frequency of repeats of very similar amino acids. Analysis of structures in the Protein Data Bank revealed that a higher degree of disorder in a protein secondary structure is associated with more perfect repeats and that the conformational disorder of IDPs depends mainly on repetitive, low-complexity sequences with limited hydrophobicity.^55−58^

IDP proteins do not have a well-defined three-dimensional structure but rather a large number of accessible, distinct conformations resulting from chain–chain and chain–solvent interactions. IDPs that prefer chain–solvent interactions adopt an extended/spiral conformation. In contrast, proteins that prefer chain–solvent interactions adopt a compact/globular conformation. When these two effects balance each other, the result is a heterogeneous, often asymmetric structure.^55,58,59^ Therefore, this large ensemble of conformations must be described in a statistical manner using the gyration radius *R*_g_ or the hydrodynamic radius *R*_h_, which provide a rough measure of the compactness of the protein and allow comparison of proteins of similar lengths.^60,61^

The DLS results in Figure 7 show that both HUG and HELP biopolymers undergo an almost instantaneous conversion from single chains to larger particles upon an increase in temperature. From Figure 5, it can be seen that the kinetics of coacervation/dissolution of HUG is different from that of HELP. The return of absorbance to baseline values during cooling indicates that the phase separation process is largely reversible for the protein HELP. In contrast, the observed hysteresis or nonreversible behavior in the turbidity profiles of the HUG solutions suggests that stable, even giant aggregates form during coacervation and remain stable upon cooling. The disassembly process does not appear to be as rapid as self-assembly, so the number of HUG large particles that remain below the coacervation temperature upon cooling is high.

Fluorescence spectroscopy is a sensitive and very practical method for evaluating binding events. Because the intensity and wavelength are very sensitive to the change in environment caused by ligand binding, the variation in fluorescence intensity as a function of ligand concentration provides information about the strength of the protein–ligand interaction. BR has two chromophores called exo- and endo-chromophores (vinyldipyrrinone), which have the same chemical formula but differ slightly in structure.^5,62^ The relative orientations and distances between the exo- and endo-chromophores are determined by the flexibility of the BR structure with respect to the two dihedral (rotation) angles. Due to the relative free dihedral rotations and flexibility of BR, efficient nonradiative decay of the excited BR in solution results in very weak fluorescence emission. When UnaG binds tightly to BR, the two chromophores are much more aligned around BR due to constraints arising from the interaction of the neighboring amino acid residues in the protein, which increases its conformational rigidity.^5^

The final goal of this work was to evaluate the influence of the HELP domain on the BR-binding properties of the UnaG domain in HUG. The *K*_d_ value determined in the presence of BSA for HUG (1.1 × 10^–9^ M) was about 10 times higher than that determined for UnaG (0.098 × 10^–9^ M, Kumagai and co-workers; 0.031 × 10^–9^ M, Shitashima and co-workers) (Table 7).^5,21^

**Table 7:** Binding Constant for BR-HUG Complex Formation in PBS Solutions and Comparison of Our Data to Those Found in the Literature for Similar Binding Processes

	*K*_d_, M	*K*_a_, M^–1^	analytical technique	references
BR + UnaG	0.098 ×·10^–9^	10 ×·10^9^	fluorescence	(5)
BR + UnaG	0.031 ×·10^–9^	32 ×·10^9^	fluorescence	(21)
BR + HUG	1.7 ×·10^–9^	0.59 ×·10^9^	fluorescence	(6)
BR + HUG + BSA 4 g·L^–1^	1.1 ×·10^–9^	0.91 ×·10^9^	fluorescence	this work
BR + HSA	87 ×·10^–9^	0.012 ×·10^9^	UV–vis	(5)
BR + BSA	45 ×·10^–9^	0.022 ×·10^9^	fluorescence	(63)
	37 ×·10^–9^	0.027 ×·10^9^	UV–vis	(41)
	83 ×·10^–9^	0.012 ×·10^9^	fluorescence	(42)

However, this value is still lower than that of serum albumin (*K*_d_ = 10^–7^- 10^–8^ M, from Chen *et al.*, Faerch *et al.*, Petersen *et al.*, Williams *et al*).^41,42,63,64^ It should be noted that HUG also tends to chain associate at temperatures below the ITT, as shown by DLS. However, the interactions due to the HELP domain do not limit the accessibility of the UnaG domain to BR in albumin solution as a 1:1 stoichiometry of bilirubin—HUG was observed by fluorometric measurements.

The differences between the binding constants of BR with respect to HUG and albumin proteins explain the displacement of BR from albumin by a competitive process due to the UnaG domain in HUG.^5,65^ HUG showed lower affinity for bilirubin than UnaG, suggesting that the intermolecular interactions in HUG aggregates affect the bilirubin binding site. However, there was a displacement of bilirubin from bovine serum albumin to HUG, which justifies the purpose of analyzing bilirubin in animal blood.

## 5 Conclusions

This study shows that HELP is a good platform for the synthesis of new fusion proteins with tailored functional domains.

The scattering results indicate that the HUG solubilization process can be optimized by first solubilizing the protein at room temperature and then cooling it, resulting in consistently higher protein yields.

The microaggregates in solution detected by scattering measurements do not alter the binding capacity of the UnaG domain, as confirmed by fluorometric analyses. This evidence may support the possibility of using UnaG in a hydrogel obtained by crosslinking the HELP domain.

This study quantifies the binding capacity of HUG in the presence of albumin using the fluorometric technique and provides us with the basis for improving the analytical method to quantitatively determine the concentration of BR in biological samples at the nanoscale. HUG is a useful bifunctional polypeptide that retains the key physicochemical properties of its individual domains (HELP and UnaG), albeit with different quantitative parameters. The versatility of HUG can be exploited to analyze BR in animal fluids via the UnaG domain, which proves to be a powerful probe for the detection of BR even in fusion proteins and opens the possibility of using the biopolymer in a broader technological setting due to its HELP domain.

## Supporting Information Available

Quality evaluation of HUG lot; CD spectra of HUG and HELP; Scatchard plot of the HUG-BR complex; Debye plot of HUG and HELP; and DLS correlation coefficient and size distribution of proteins (PDF)
