# Supplementary material for: Macromolecular and Solution Properties of the Recombinant Fusion Protein HUG
Source: Biomacromolecules. 2022 Jul 25;23(8):3336–48. doi: 10.1021/acs.biomac.2c00447 (PMC9364316; doi:10.1021/acs.biomac.2c00447)

**Supporting Information for publication**

# Macromolecular and solution properties of the recombinant fusion protein HUG

Paola Sist[1], Antonella Bandiera[1], Ranieri Urbani[2*], Sabina Passamonti[1]

[1]Department of Life Sciences, University of Trieste, via Giorgieri 1, I-34127, Trieste, Italy.

[2]Department of Chemical and Pharmaceutical Sciences, University of Trieste, via Giorgieri 1, I-34127, Trieste, Italy.

**Figure S1**. Characteristics of the HUG lot used in this study.

(a) Absorption spectra (ABS) of HUG (—) and HELP (---) solutions (2 g·$L^{-1}$) were recorded in quartz cells (d = 1 cm) using a double-beam spectrophotometer (CARY-4E UV-visible spectrophotometer) at room temperature.

(b) SDS-PAGE separation of HELP (lane 1) and HUG (lane 3) (4 μg in each lane). The standard molecular weight is loaded in lane 2.

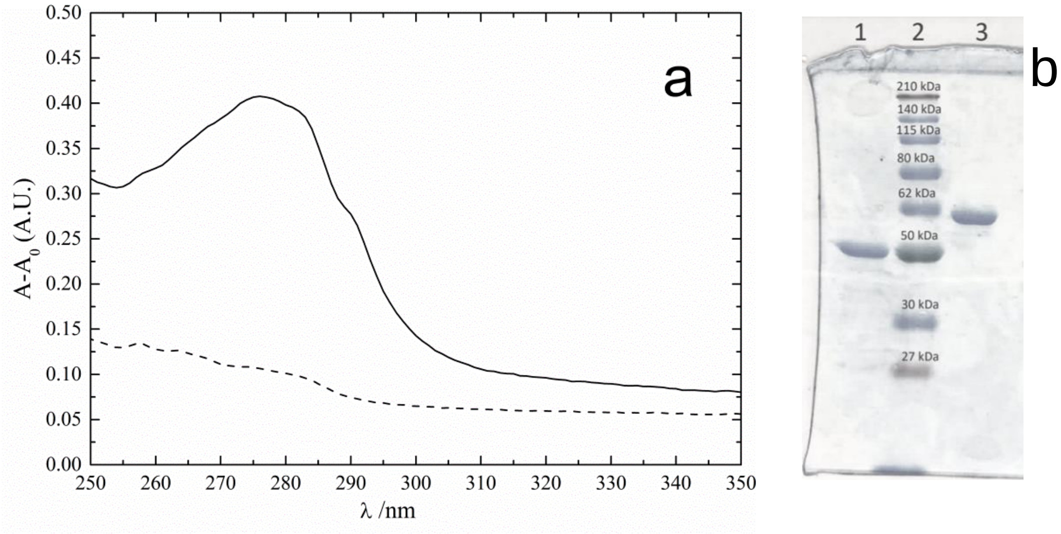

**Figure S2**. CD spectra of HUG (a) and HELP (b) without and with BR in the nanomolar range.

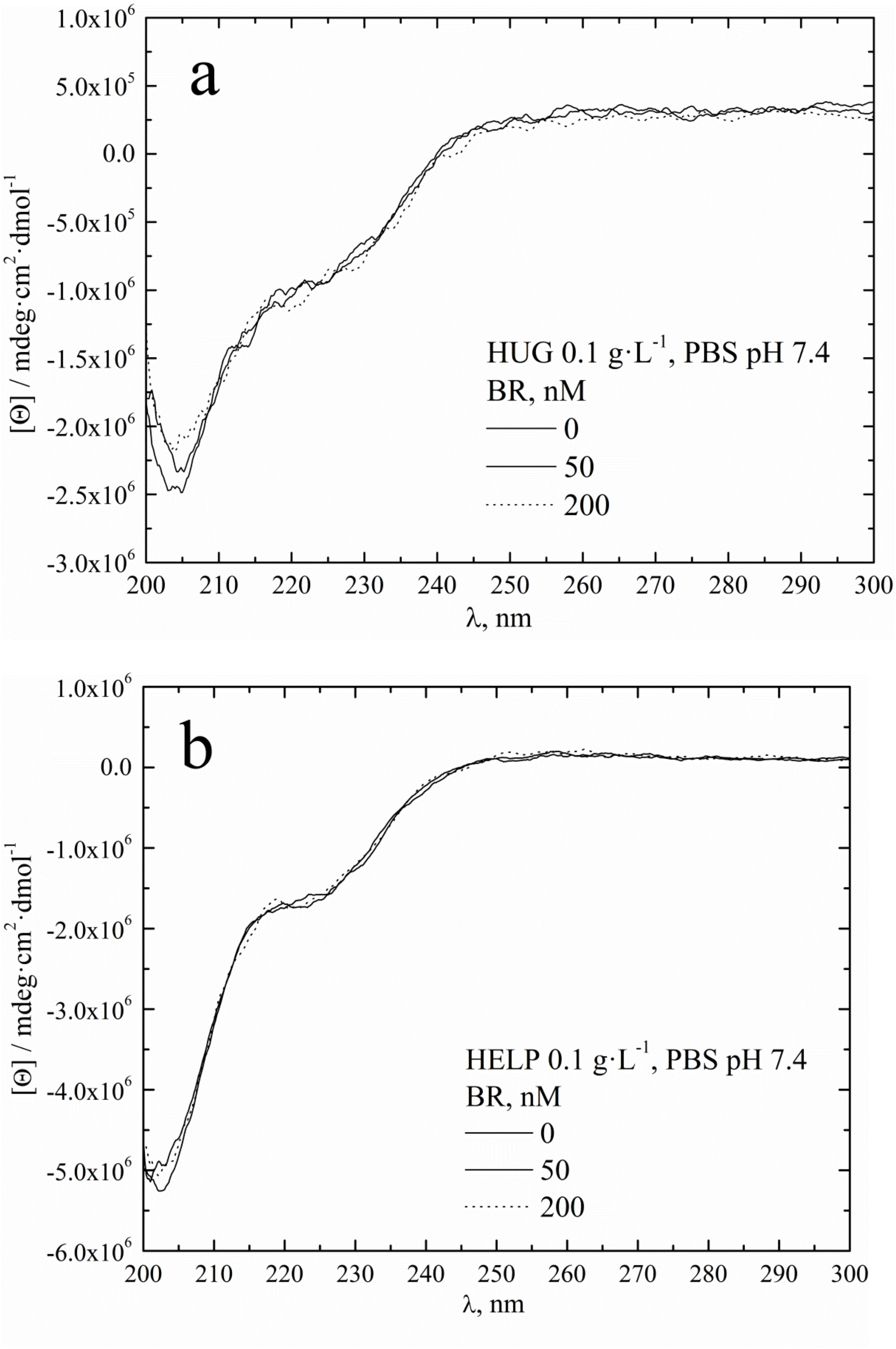

**Figure S3**. Scatchard plot of HUG-BR complex.

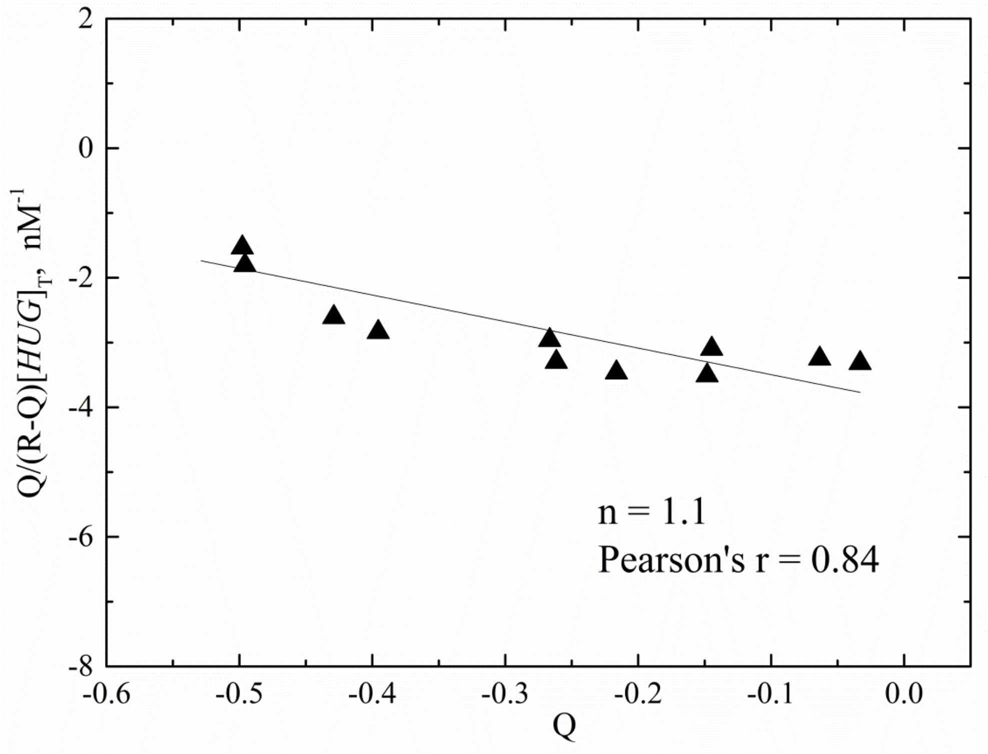


Tha Scatchard plot was obtained by using equation (Levine, 1977):

$$\frac{Q}{(R-Q)[HUG]_T} = nK_a - K_a Q$$

where: $$Q = \frac{[BR\!-\!HUG]}{[HUG]_T}$$

$$R = \frac{[BR]_T}{[HUG]_T}$$

with $[HUG]_T$ and $[BR]_T$ the total concentration of HUG and BR, respectively.

Following Levine (1977), Q is related to the fractional saturation $Y=F/F_0$ as defined above. The linear fitting of data in Figure S3 gives n = 1.1.

**Figure S4**. One representative Debye plot of HUG and HELP in solution from Static Light Scattering measurements.

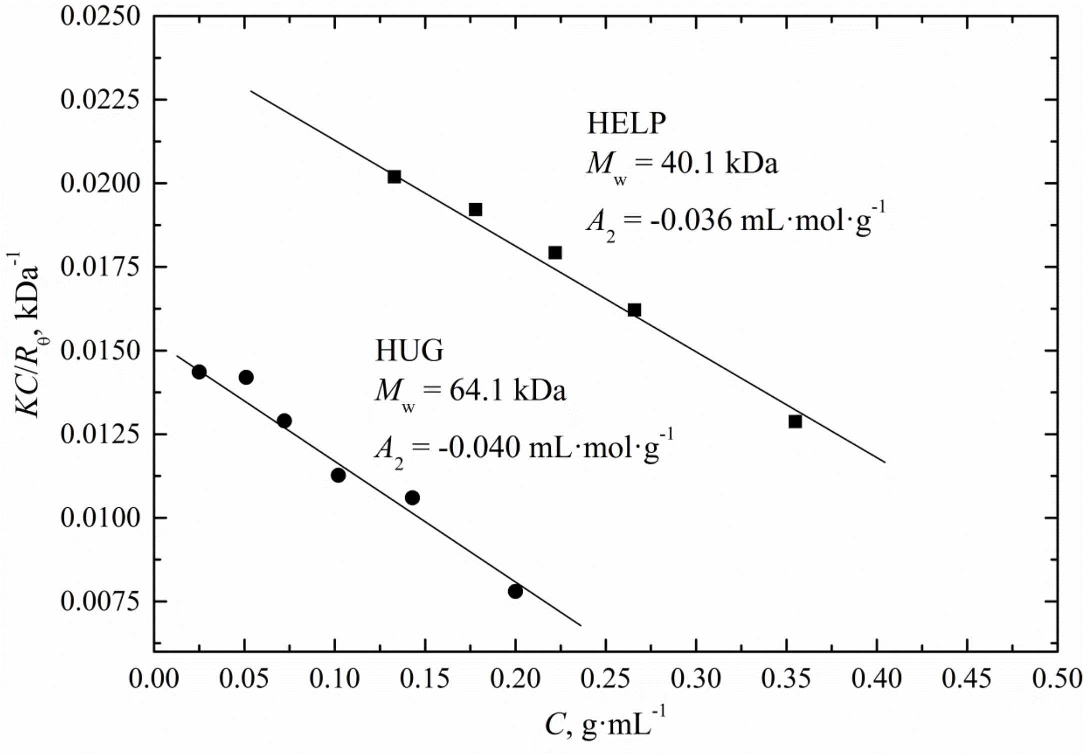

**Figure S5**. DLS Correlation coefficient (**a**) and size distribution (by intensities) (**b**) of HELP protein at T=30°C, C=0.1 g·L$^{-1}$.

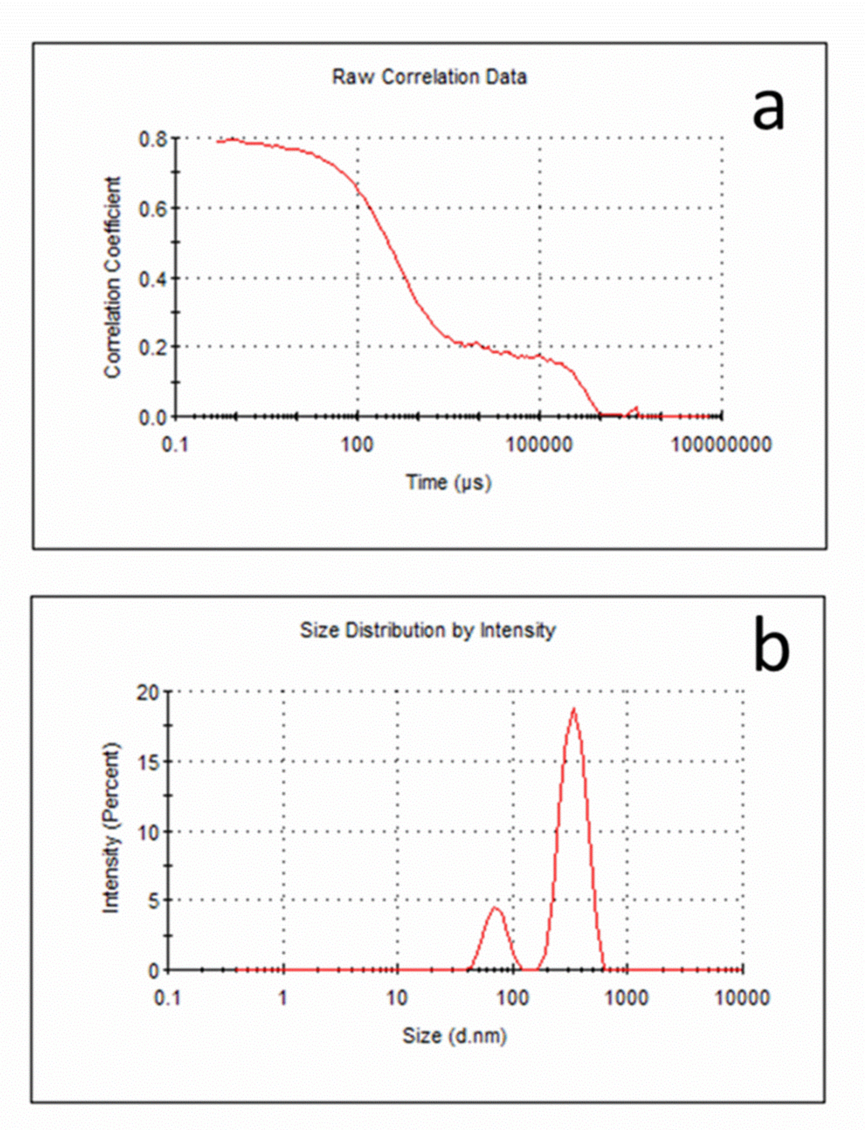

**Figure S6**. DLS Correlation coefficient (**a**) and size distribution (by intensities) (**b**) of HUG protein at T=30°C, C=0.1 g·L$^{-1}$.

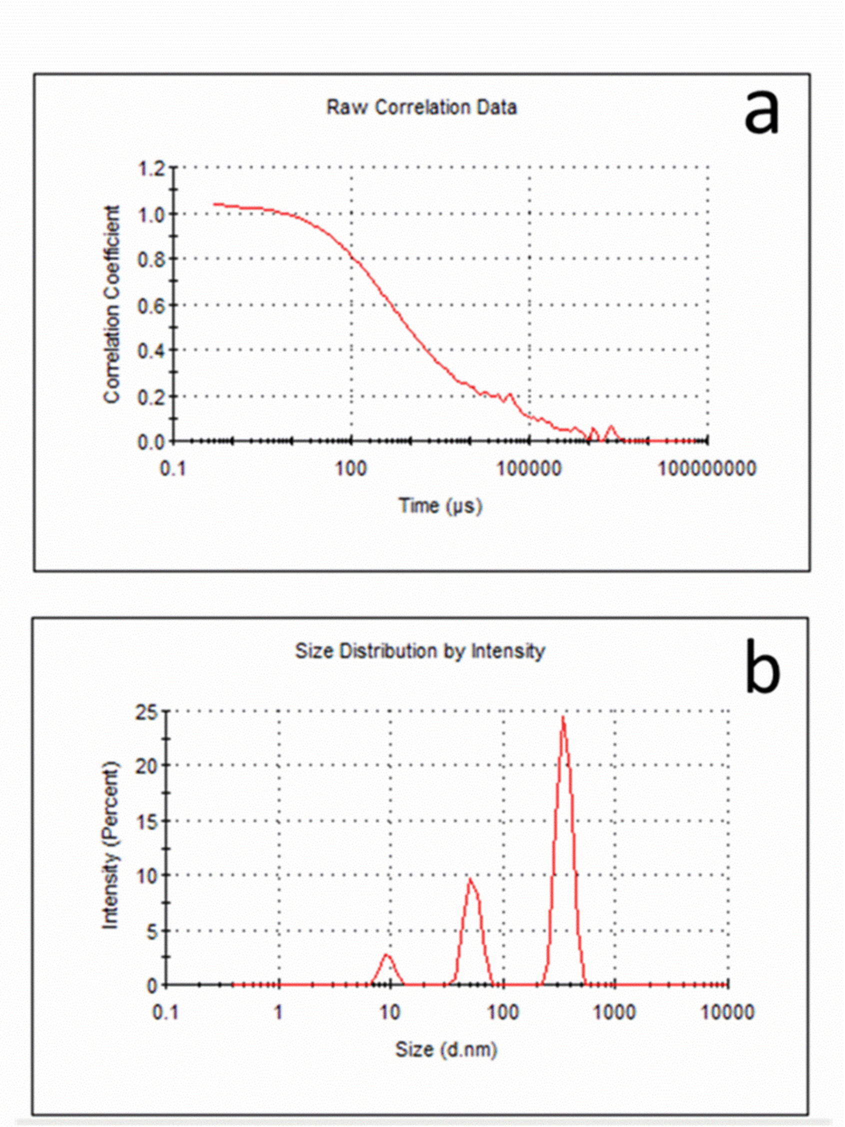

Supplement: Supplementary file 1 — bm2c00447_si_001.pdf [file bm2c00447_si_001.pdf]
